# Sphingolipids as a Novel Therapeutic Target in Radiation-Induced Lung Injury

**DOI:** 10.1007/s12013-021-01022-8

**Published:** 2021-08-09

**Authors:** Jeffrey R. Jacobson

**Affiliations:** Department of Medicine, Division of Pulmonary, Critical Care, Sleep and Allergy, University of Illinois at Chicago, Chicago, IL USA

**Keywords:** Radiation lung injury, Endothelial cells, Sphingosine-1-phosphate, Statins, UCHL1

## Abstract

Radiation-induced lung injury (RILI) is a potential complication of thoracic radiotherapy that can result in pneumonitis or pulmonary fibrosis and is associated with significant morbidity and mortality. The pathobiology of RILI is complex and includes the generation of free radicals and DNA damage that precipitate oxidative stress, endothelial cell (EC), and epithelial cell injury and inflammation. While the cellular events involved continue to be elucidated and characterized, targeted and effective therapies for RILI remain elusive. Sphingolipids are known to mediate EC function including many of the cell signaling events associated with the elaboration of RILI. Sphingosine-1-phosphate (S1P) and S1P analogs enhance EC barrier function in vitro and have demonstrated significant protective effects in vivo in a variety of acute lung injury models including RILI. Similarly, statin drugs that have pleiotropic effects that include upregulation of EC S1P receptor 1 (S1PR1) have been found to be strongly protective in a small animal RILI model. Thus, targeting of EC sphingosine signaling, either directly or indirectly, to augment EC function and thereby attenuate EC permeability and inflammatory responses, represents a novel and promising therapeutic strategy for the prevention or treatment of RILI.

## Introduction

Radiation-induced lung injury (RILI) represents a dose-limiting toxicity associated with thoracic radiotherapy for which effective treatments remain non-existent. RILI encompasses a clinical spectrum of disease manifested early (6–12 weeks after radiation) by pneumonitis and later (at 6–12 months) by fibrosis associated with irreversible impairment of lung function [1]. Reports of the incidence of RILI after radiotherapy are highly variable due to differences amongst patient populations at risk including the underlying disease, the specific radiation protocols administered, and the existence of any number of co-morbidities known to contribute to risk [2]. In addition, variable incidences of RILI are associated with variable radiation treatment parameters including the percentage of total lung volume dose, the mean lung dose, and the normal tissue complication probability derived from the lung dose–volume histogram [3]. Nonetheless, one study of over 800 patients treated with radiotherapy for non-small cell lung cancer found an overall incidence of symptomatic pneumonitis of 30% with fatal pneumonitis in 1.9% [4], numbers corroborated by a smaller study published near the same time [5]. Thus, the lack of effective RILI treatment represents an important, unmet medical need and discovery of effective therapies would yield significant clinical benefits to a wide range of potential patients.

## Targeting Sphingolipids as a Strategy to Enhance Lung Vascular Integrity

Lung inflammatory syndromes, including RILI, represent a collection of clinical conditions of which a key feature is endothelial dysfunction and increased vascular permeability. The clinical consequences of these changes are impaired oxygenation and lung function associated with non-uniform radiographic changes. These characteristic changes in RILI support endothelial cell (EC) barrier dysfunction as a key determinative event, consistent with other forms of acute lung injury (ALI). Accordingly, strategies aimed at augmenting or restoring endothelial barrier function offer therapeutic promise and potential. Our lab and others have previously identified specific molecular targets including sphingolipids, as particularly promising in this regard [6–9].

Sphingolipids, constituents of cell plasma membranes, mediate a variety of cell signaling events. Sphingosine-1-phosphate (S1P) and ceramide are two sphingolipids that have been implicated as mediators of inflammatory lung injury in response to radiation. While S1P is a pro-survival molecule, ceramide promotes apoptosis and its production is increased in response to cellular stress including that caused by radiation [10]. S1P is derived from the phosphorylation of sphingosine via sphingosine kinase (SphK), while the acylation of sphingosine produces ceramide. In turn, ceramide coupling to phosphocholine results in sphingomyelin. Conversely, ceramide can be produced from sphingomyelin by sphingomyelinases of which there are several isoenzymes including acid sphingomyelinases (ASMase) [11]. ECs are rich in ASMase and ASMase measured in the serum or directly in the lung have been found to be increased in animal models of inflammatory lung injury [12]. In addition, increased lung vascular permeability has been linked to ASMase-dependent production of ceramide in murine ALI [13].

Five G protein-coupled S1P receptor subtypes have been identified and are widely expressed on various cell types. S1PR1, S1PR2, and S1PR3 are predominantly expressed on EC but are coupled to different G proteins resulting in distinct downstream signaling effects [14]. S1PR1 ligation by S1P promotes EC barrier integrity through Rac1-mediated augmentation of the cortical actin cytoskeleton. The therapeutic potential of S1P in acute inflammatory lung injury syndromes was initially suggested by S1P-induced EC barrier enhancement in vitro associated with increased EC barrier function [7, 15].

Subsequently, S1P was found to be protective in pre-clinical ALI models as well [16, 17]. In addition, differential effects have been reported with respect to lung vascular permeability in response to S1P, the structurally similar compound, FTY720, and various S1P analogs [9]. These studies confirmed the (S)-phsophonate analog of FTY720, tysipinate, as having particularly robust effects on EC barrier protection.

## Sphingolipids as Mediators of Radiation-Induced Lung Injury

Our group has thoroughly characterized a murine RILI model that we have employed to study potential therapeutic strategies [18–21]. Briefly, animals are administered a single dose of thoracic radiation (10–25 Gy) and are then assessed at various time points up to 6 weeks afterwards when RILI is fully realized with significantly increased interstitial inflammatory cells appreciable on lung histology and marked increases in protein, cell counts, and inflammatory cytokines measurable in bronchoalveolar lavage fluid (BALF). We have confirmed the central involvement of sphingolipids in our RILI model as evidenced by differential expression of sphingolipid components in response to radiation in plasma, BALF, and lung tissue [19]. We have also reported differential RILI responses in SphK1 knockout mice and mice with reduced expression of specific S1P receptors as well as the protective effects S1P signaling augmentation. This work has led to the study of a variety of molecules that have become critical to our understanding of sphingolipid-mediated RILI pathogenesis including macrophage inhibitory factor [21] and growth arrest and DNA damage repair gene 45 alpha (GADD45a) [20]. Moreover, as we have reported increased RILI susceptibility of GADD45a–/– mice and identified decreased expression of ubiquitin c-terminal hydrolase L1 (UCHL1), a deubiquitinating enzyme, as a mediator of increased lung injury in these animals. We have also now linked these observations to effects on sphingosine pathway components [20, 22, 23].

Thus, the targeting of sphingolipid signaling as a therapeutic strategy to prevent or mitigate RILI can be broadly grouped into either direct or indirect approaches. The use of S1P or S1P analogs is indicative of approaches that directly augment sphingolipid pathway signaling. Alternatively, a variety of different strategies can be considered to indirectly augment sphingolipid signaling, for example, via upregulation of S1PR1 or modulation of SphK expression levels. These ideas are discussed in more detail below.

## Therapeutic Potential of Direct Targeting of Sphingolipid Pathways in RILI

### **RILI Protection by S1P Analogs**

Several reports have confirmed robust protective effects of S1P in animal models of ALI. However, the translational values of these observations are limited but serious safety concerns related to the use of S1P in patients. Specifically, S1P administration is associated with potential cardiovascular effects including significant bradycardia as well as reductions in cardiac output [24, 25]. Accordingly, interest in S1P analogs, which similarly confer vascular protection but may have more favorable safety profiles, led to the study of these molecules in our RILI model. These include FTY720, which admittedly has safety concerns of its own, as well as SEW-2871, an S1PR1 agonist, and tyspinate, a phosphonate S1P analog, all three of which have demonstrated protective effects in pre-clinical ALI models [9, 16, 26, 27].

**Fig. 1:**
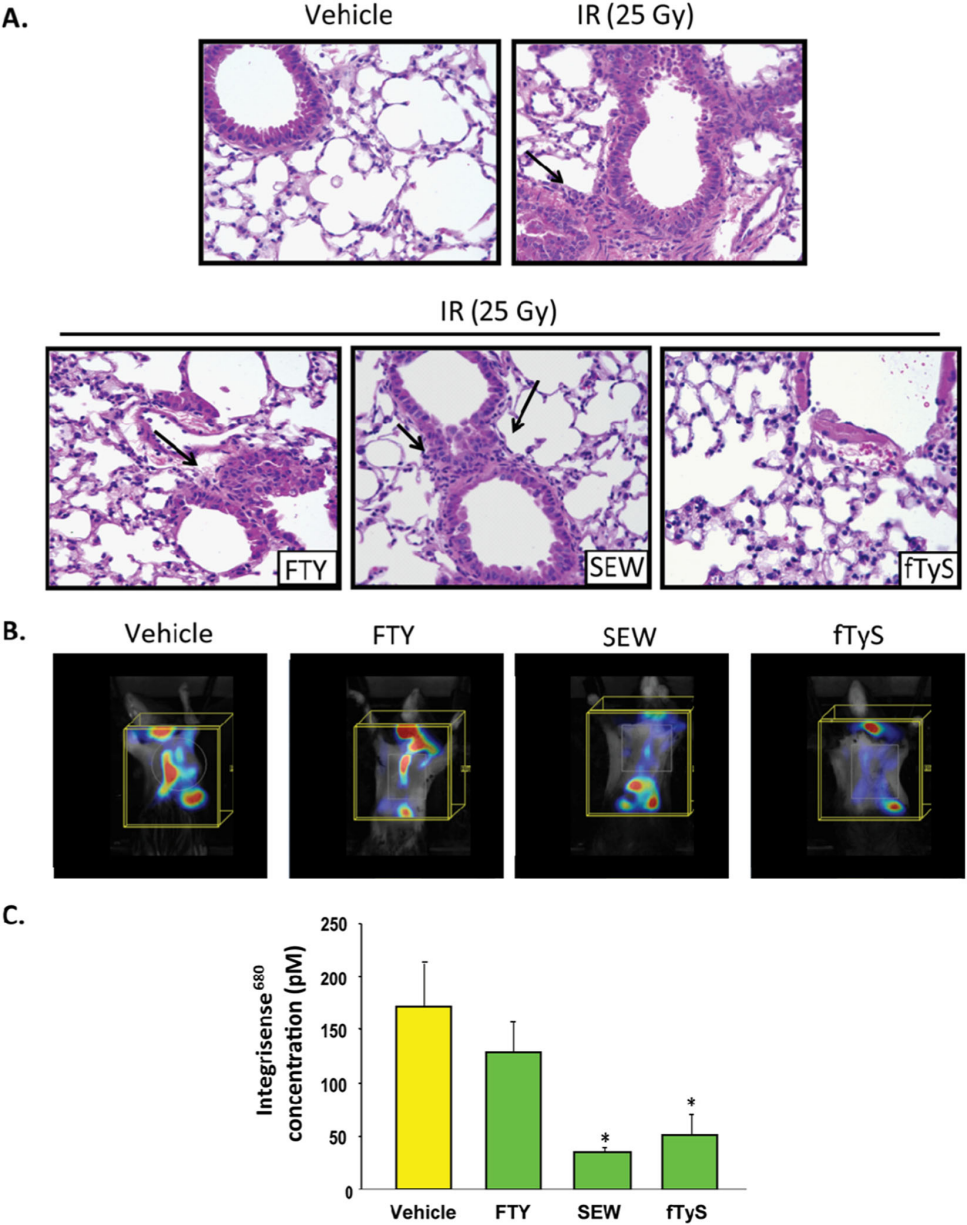
Protective effects of S1P analogs in murine RILI. **A** Hematoxylin and eosin staining of lung sections from mice administered a single dose of thoracic radiation (25 Gy) demonstrate modest interstitial edema but a prominent influx of inflammatory cells (arrow) at 6 weeks compared with that in uninjured controls. These changes are visibly attenuated in RILI-challenged mice treated with SEW or tyspinate (fTyS, 0.1 mg/kg intraperitoneal injection, administered 2×/weeks beginning 1 weeks before irradiation). In contrast, lungs from RILI-challenged mice treated with FTY720 (0.1 mg/kg) were not significantly different in appearance at 6 weeks from lungs of mice subjected to radiation alone. **B** In separate experiments, RILI-challenged mice (25 Gy) were injected with an intravascular probe (IntegriSense 750) 6 weeks post radiation and then subjected to VisEn FMT imaging 6 h later (shown from left to right: vehicle, FTY, SEW, tyspinate). **C** SEW and tyspinate (fTyS) treatment (0.1 mg/kg) significantly decreased radiation-induced dye extravasation. There was no evidence of protection in animals treated with FTY720 (0.1 mg/kg) compared with RILI controls. Reprinted with permission [19].

In these studies, agonists were given to mice prior to being subjected to single dose thoracic radiation (20 Gy) with lung injury assessed at 6 weeks via BALF analyses. Notably, tyspinate had the most dramatic effect with both low and high dosing (0.01 or 0.1 mg/kg) resulting in a significant attenuation of RILI. In comparison, SEW was found to be protective with higher dosing (0.1 mg/kg) while no effect was observed with FTY720. Furthermore, evidence of protection was observed with all three agonists as assessed by lung histology as well as by VisEn FMT lung imaging (Perkin Elmer, Bedford, MA). This technique allows for real-time imaging of microvascular leak in vivo utilizing a fluorescence imaging agent (IntegriSense 750 NIR, Perkin Elmer) that targets the vasculature as a selective nonpeptide small molecule integrin α_v_β_3_ antagonist and a near-infrared fluorochrome. Animals were injected with the probe 6 weeks post radiation and imaged 24 h later (Fig. 1).

While these experiments were largely proof of principle, they support the idea that S1P analogs could potentially be used to prevent or treat RILI. Importantly, treatment with these S1P analogs was well tolerated by the animals that received them with no adverse effects appreciated. Moreover, with respect to the potential clinical applications, unlike other ALI patients whose disease is largely unpredictable, the idea of pre-treatment strategies could easily be adapted and applied to patients undergoing radiation therapy, particularly those who may be deemed to be at increased risk for RILI.

## Therapeutic Potential of Indirect Targeting of Sphingolipid Pathways in RILI

### Statins

The statin drugs are a class of HMG CoA-reductase inhibitors that have been widely used for their ability to lower serum cholesterol levels and for their beneficial effects in patients with or at high risk for cardiovascular disease. These drugs, however, are also recognized to have pleiotropic effects independent on their lipid-lowering properties. Included amongst these are direct effects on EC signaling and vascular function. Arguably, with respect to effects just on EC alone these drugs could also said to have pleiotropic properties via inhibition of protein prenylation including effects on RhoGTPase activation, NADPH oxidase activation, and nitric oxide expression, as well differential expression of a variety of genes including mediators of actin cytoskeletal dynamics and inflammation, determinants of EC barrier function and vascular permeability, as well as effects on sphingolipid pathway components [28–31]. Indeed, the relevance of these effects to vascular permeability in vivo is supported by statin protection in a variety of small animal models of ALI [29, 32–34].

With respect to sphingolipids in particular, we previously reported that simvastatin upregulates S1PR1 in human lung ECs via increased expression of Krüppel-like factor 2 (KLF2), a transcription factor regulator of EC stress responses [35]. Simvastatin induces increased *S1PR1* promoter activity that is associated with KLF2 recruitment to the *S1PR1* promoter. Furthermore, this activation of the *S1PR1* promoter is attenuated by KLF2 silencing (siRNA). The functional importance of these effects is evidenced by augmentation of S1PR1-mediated EC barrier augmentation by simvastatin that is reduced by KLF2 silencing as measured by changes in transendothelial electrical resistance [35].

**Fig. 2:**
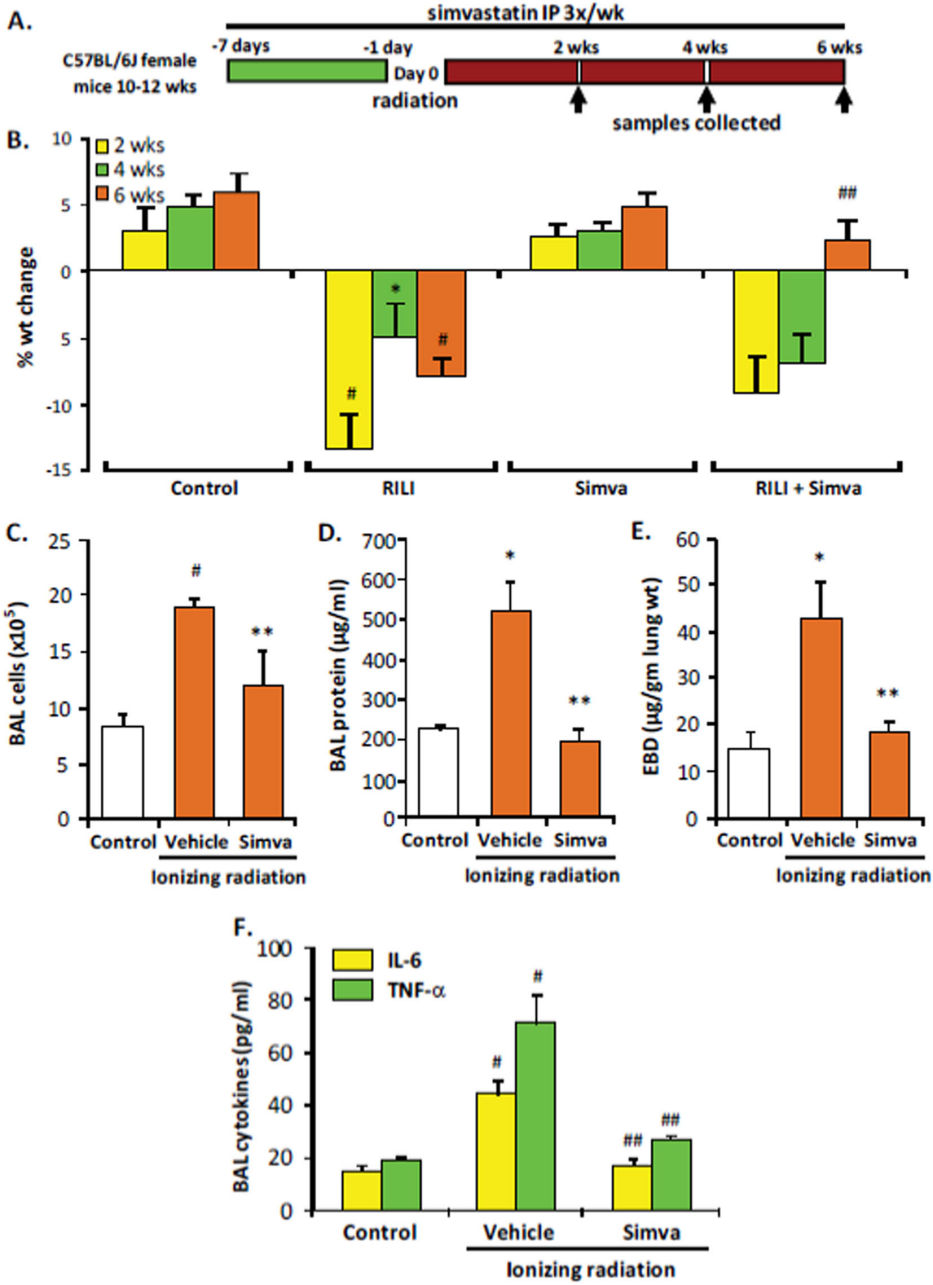
Protective effects of simvastatin in murine RILI. **A** Mice received simvastatin (10 mg/kg body weight, 3×/weeks) or vehicle beginning 1 week prior to radiation (25 Gy, single dose) and continuing up to 6 weeks post irradiation with collection of BALF and tissue at the intervals indicated. **B** Simvastatin treatment of radiated mice resulted in significantly increased body weight at 6 weeks compared to radiated controls (*n* = 5 animals/group; ^##^*p* < 0.01 compared to RILI alone) while radiation exposure alone produced significant weight loss at 2, 4, and 6 weeks (*p < 0.05 and ^#^*p* < 0.01 compared to controls). In addition, simvastatin attenuated radiation-induced increases in BAL cell counts (**B**) and both BAL protein (**C**) and Evans Blue Dye (EBD) extravasation (**E**) at 6 weeks (*n* = 5 animals/group, *p < 0.05 and ^#^*p* < 0.01 compared to controls; **p < 0.05 compared to RILI alone). **F** Earlier, at 4 weeks post irradiation, simvastatin treatment was associated with a significant reduction in BALF TNF-α and IL-6 (*n* = 5 animals/group, ^#^*p* < 0.01 compared to controls and ^##^*p* < 0.01 compared to radiation alone). Reprinted with permission [18].

In our murine RILI model, statin treatment was also found to be protective [18]. In these studies, mice were pretreated with simvastatin 10 mg/kg via intraperitoneal injection 3×/week beginning 1 week prior to single dose (25 Gy) thoracic radiation and continued treatment until 6 weeks after irradiation when lung injury was assessed. These experiments confirmed robust protection associated with simvastatin treatment as measured by BALF protein, cell counts, and inflammatory cytokines (Fig. 2). In addition, lung histology confirmed decreased RILI-associated inflammatory cell infiltration by simvastatin and decreased lung vascular leak was confirmed by measurements of Evans Blue dye extravasation and, separately, by VisEn FMT lung imaging.

While there are multiple potential mechanisms underlying RILI protection by simvastatin, evidence suggests that the known effects on sphingolipid signaling are an important factor. In support of this idea, gene expression analyses from these studies identified relevant radiation-induced dysregulation of lung gene expression by statins as well as overrepresented canonical pathways that were both dysregulated by irradiation and differentially effected by simvastatin treatment that included sphingolipid pathway genes.

### UCHL1

As previously noted, we have identified deubiquitination by UCHL1, a deubiquitinating enzyme, as an important mediator of murine RILI. This finding stems from the identification of GADD45a as a novel candidate gene in pre-clinical models of ALI [36]. Subsequently, we found that GADD45a–/– mice express decreased Akt and increased Akt ubiquitination as a consequence of increased DNA methylation resulting in reduced UCHL1 expression [22] and we confirmed that GADD45a–/– mice are more susceptible to RILI-challenge [20]. Moreover, evidence for a possible mechanistic link between UCHL1 and sphingolipid signaling in RILI is suggested by the known polyubiquitination of SphK1 previously reported by Natarajan et al. [37].

To further explore the potential role for UCHL1 in RILI, we assessed UCHL1 expression levels from whole lung homogenates of RILI-challenged mice and found significant increases in UCHL1 mRNA and protein relative to controls. In addition, we confirmed that UCHL1 knockdown (siRNA) has a detrimental effect on EC barrier function as measured by transendothelial electrical resistance changes over time in response to both barrier-enhancing and barrier-disruptive agonists. Subsequently, we used a pharmacologic UCHL1 inhibitor, LDN-57444, and confirmed augmented RILI associated with UCHL1 inhibition. Finally, in separate experiments, EC SphK1 levels were inversely affected by UCHL1-silencing and overexpression while inhibition of SphK1 resulted in decreased basal and radiation-induced EC UCHL1 expression [38].

These findings strongly support the idea that SphK1 ubiquitination by UCHL1 is an important determinant of the elaboration of RILI and suggest that strategies aimed at increasing UCHL1 activity could confer RILI protection. To this end, molecules that have been identified to be novel UCHL1 potentiators are particularly intriguing and warrant study in RILI models [39].

## Conclusion

There is no question that RILI represents an area of clinical medicine for which current treatments are sorely lacking and for which targeted therapies are non-existent. Accordingly, consideration of previously under-recognized mechanisms that drive RILI pathobiology is warranted in the hope of identifying novel and effective therapies. The sphingolipid pathway certainly merits study in this regard and mounting evidence suggests that direct targeting of sphinglipid signaling with S1P and its analogs could be highly effective for the treatment or prevention of RILI. Moreover, while S1P and several analogs have been studied and shown promise in RILI models, several other analogs have yet to be investigated in this context including CYM-5442, AUY954, and ponesimod, three other S1PR1 agonists [40–42].

Separately, while statin drugs and strategies aimed at augmenting UCHL1 activity are both promising RILI therapies, these are only two examples of possible approaches intended to indirectly augment the EC-protective effects of sphingolipid signaling. Undoubtedly, there are countless other therapies that could leverage this same idea and would warrant their own investigation in this context in an effort to address this unmet medical need. As the role of sphingolipids in RILI pathobiology continues to be defined and becomes more widely recognized, it is virtually certain that related advances will be made that translate directly to changes in patient care practices for those with or at risk for RILI.

## Abbreviations

ALI: acute lung injury
ASMase: acid sphingomyelinases
BALF: bronchoalveolar lavage fluid
EC: endothelial cell
GADD45a: growth arrest and DNA damage repair gene 45 alpha
KLF2: Krüppel-like factor 2
RILI: radiation-induced lung injury
SphK: sphingosine kinase
S1P: sphingosine-1-phosphate
S1PR1-3: sphingosine-1-phosphate receptor 1-3
UCHL1: ubiquitin c-terminal hydrolase L1
